# Head and Neck Trauma in a Rapidly Growing African Metropolis: A Two-Year Audit of Hospital Admissions

**DOI:** 10.3390/ijerph16244930

**Published:** 2019-12-05

**Authors:** Irene Kida Minja, Michael Lowery Wilson, Masood Ali Shaikh, Leila Perea-Lowery

**Affiliations:** 1 School of Dentistry, Muhimbili University of Health and Allied Sciences, P.O. Box 65014, Dar es Salaam, Tanzania; ikminja@gmail.com; 2 Injury Epidemiology and Prevention Research Group, Turku Brain Injury Centre, Turku University Hospital and University of Turku, 20521 Turku, Finland; masoodalishaikh@gmail.com (M.A.S.); leila.perea@utu.fi (L.P.-L.); 3 Heidelberg Institute of Global Health, University of Heidelberg, 69120 Heidelberg, Germany

**Keywords:** urban, demographics, injury epidemiology, global health, maxillofacial trauma

## Abstract

Understanding injury-related burdens is an essential part of trauma quality improvement programs aimed at decreasing morbidity and mortality. This is especially the case in low and middle-income country settings where data on injuries remains limited. The aim of this study was to audit the types of head and neck injuries, which have been diagnosed among patients admitted to a major national hospital in the context of a rapidly growing sub Saharan city. Data were collected retrospectively for head and neck trauma from the Muhimbili National Hospital (MNH) in Dar es Salaam, Tanzania from the years 2016 and 2017. Distribution of ICD-10 codes by age and sex for the five most common diagnoses were determined using frequencies and percentages. The most common diagnosis was ICD-10-S02 (fracture of skull and facial bones) with 277 cases (44.1%), which was followed by S05 (injury of the eye and orbit), 114 cases (18.2%), and S09 (other and unspecified injuries of head) 77 cases (12.3%). The mean ages of admission for these three diagnoses were 28.1 (SD: 11.6), 23.8 (SD: 18.9), and 30.8 (SD: 18.0) years, respectively. This study provides information on the overall burden of head and neck trauma at a major regional tertiary care facility. It provides an initial understanding of the burden of head and neck trauma and suggests follow-up in the form of clarification of injury mechanisms and contextual factors for future work.

## 1. Introduction

Out of the top 10 causes of morbidity and mortality in low and middle-income countries (LMICs), traumatic injuries consistently rank among the top three [1]. By 2020, seven out of every ten deaths are expected to be due to non-communicable diseases in LMICs, with injuries expected to overtake communicable diseases as a main source of morbidity [2]. As injuries surpass communicable diseases as the leading causes of morbidity and mortality, their increasing burdens will have devastating effects on the individual victims; their families and will overburden already fragile health systems [3]. Currently, traumatic injuries account for more than 5 million deaths every year worldwide—the same amount as HIV, malaria, and tuberculosis combined [4]. Prevention efforts for injuries, despite their disproportionate societal burden, are rarely matched with the same levels of investment as their infectious disease counterparts.

Among the injuries representing the greatest threats to life and morbidity are those which affect the head and neck. These injuries can range in form, and include minor soft tissue lacerations to complex facial fractures, penetrating wounds, and cranial nerve injuries [5]. These injured areas of the head, neck, and facial regions can negatively impact masticatory function (chewing), speech, respiration, or vision with the potential for significant, life-long disability [6,7]. There is also significant risk for traumatic brain injuries (TBI) when high mechanical forces to the head and neck are such that they distort brain function [8].

Head and neck trauma are often overlooked aspects within the overall burden of injury. The focus of the vast majority of studies on head-related trauma tends to focus on those which involve insults to the brain, not recognizing that the occurrence of head and neck trauma in general remains under-documented. This paucity of information on head and neck injury is especially the case in LMIC contexts. Information on overall and specific injury burden is of great importance as a first step in the design of interventions ultimately aimed at the development of mitigation strategies.

The aim of this study was to audit the types of head and neck injuries diagnosed among patients admitted to a major regional hospital in the context of a rapidly growing sub-Saharan city.

## 2. Methods

This study was focused on Dar es Salaam, Tanzania, which has among the fastest growing urban growth rates in the world [9]. The data which informed this study were derived from the Muhimbili National Hospital (MNH) in Dar es Salaam, Tanzania for the years 2016 and 2017. Information of all patients that were admitted (inpatient) at the hospital with trauma to the head and neck region was retrieved through the Hospital management Information System—JEEVA (Jeeva Informatics Solution). At the time of diagnosis, all conditions were allocated ICD-10 codes. A data file was comprised of demographic characteristics of age and sex; date of admission in the hospital; and an ICD-10 code corresponding to the injury diagnosis.

**Figure 1:**
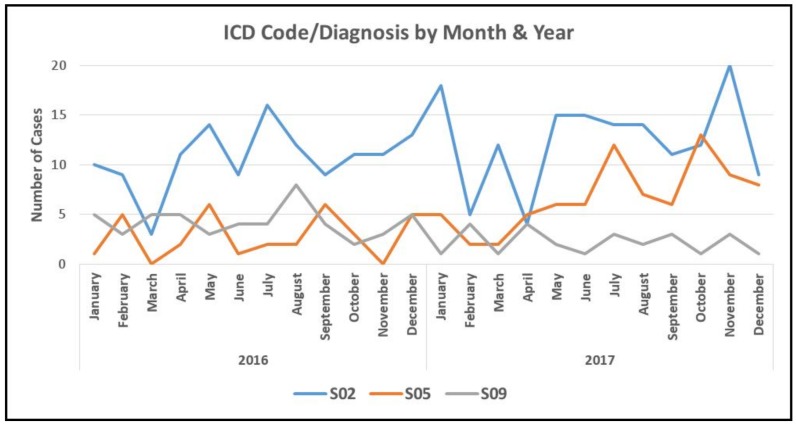
Three leading head and neck trauma diagnoses by month and year according to ICD-10 code.

The distribution of ICD-10 codes by age and sex for the five most common diagnoses were determined using frequencies and percentages. Line graphs of admissions by month and year for the three common diagnoses were created (Figure 1). Additionally, associations with age and sex for the most common three diagnoses were statistically determined using the chi-square test.

The Muhimbili National Hospital’s Ethics Review Board (MNH-IRB) granted the ethical approval, and permission to collect data related to this study, and to report the findings. No individual patients or their relatives were contacted during the conduct of this study.

## 3. Results

During the period under study, there were 628 patient records pertaining to head and neck trauma, which were available for examination. There were 315 cases in 2016 and 313 cases in 2017. The majority of admissions were male *n* = 510 (81.2%). The most common diagnosis was ICD-10-S02 (fracture of skull and facial bones), with 277 cases (44.1%), which was followed by S05 (injury of eye and orbit), 114 cases (18.2%), and S09 (other and unspecified injuries of head), 77 cases (12.3%). The mean ages of admission for these three diagnoses were 28.1 (range: 0.1–70), 23.8 (range: 0.8–90), and 30.8 (range: 0.1–85) years, respectively. When considering the three main diagnoses (S02, S05, and S09), significant differences existed in injury rates according to age (F = 5.57; *p* = 0.0041) and sex (chi2 = 8.72; *p* = 0.013). These results are illustrated in Table 1, Table 2 and Table 3.

Concerning Figure 1, we provide an illustration of the three most diagnosed head and neck trauma cases admitted to the Muhimbili National Hospital. Fractures of the skull and facial bones (S02) were over-represented among all admissions throughout the study period. The other prominent category of injuries was classified as “other and unspecified injuries of the head” S09. This category typically also includes injuries to the blood vessels of the head, lacerations, traumatic ruptures of the ear drum, and other injuries to tissues, tendons, and muscles which are otherwise not classified. Of the ICD-10 codes indicated in Table 3, the following are indicative of some form of intracranial injury or traumatic brain injury [10]: S02 (*n* = 277), S04 (*n* = 13), S06 (*n* = 24), and S09.9 (*n* = 50), making a total of 58% of cases, with S06 being confirmatory for traumatic brain injury in 4% of admitted patients. No monthly, seasonal, or yearly trend in head and neck trauma was noted.

## 4. Discussion

Head and neck injuries are an important health concern; however, the number of epidemiological studies conducted on head and neck injuries in Tanzania is very limited. This study provides an analysis of head and neck injuries diagnosed among patients admitted to the Muhimbili National Hospital (MNH) in Dar es Salaam, Tanzania for the years 2016 and 2017. The majority of the injuries registered were fractures of skull and facial bones. Multiple specialties are involved in the management of head and neck injuries, including general surgery, otolaryngology, plastic surgery, and oral and maxillofacial surgery. While there is little available information concerning the costs of the treatment, management, and rehabilitation associated with head and neck trauma in Tanzania, studies from high income-countries suggest that head and neck injuries are among the most costly to diagnose and treat [11,12].

In this study head and neck injuries occurred more frequently among males. This is consistent with previous research on head and neck injuries [13,14]. It is also consistent with behavioral and occupational patterns that are likely to predispose males to higher rates of head and neck injuries. For example, motorcycle users in Dar es Salaam are largely male, with many making use of them for transporting passengers as a source of income [15]. Crashes involving motorcycle riders, with or without helmets, are a major source of hospital admissions involving head and neck trauma in the region [16,17].

The age group found in this study with higher incidence of head and neck injuries was 21–35 years, which is also consistent with prior Tanzanian research on the demographic characteristics of those mostly likely to use motorcycles for transportation [15]. A study conducted in India reported similar results [18], with the highest number of victims (31%) found in the age group 20–29 years. This shows that people of the most economically productive age group are affected disproportionately by head and neck injuries, potentially having negative residual impacts on families and communities that depend on working age individuals for economic support [15,19].

While this retrospective study of clinical case reports is silent on the mechanisms and contextual factors leading to admission, other studies in the region do provide some contextual information concerning trauma in general. Authors from the Bugando Medical Center, found that head injuries were the single most common cause of trauma admissions and that these trauma admissions contributed significantly to high mortality and long-term disabilities. The majority of these admissions, the authors note, were due to motorcycle crashes [20].

In the present study, the total numbers of admitted trauma cases treated at MUHAS during 2016 and 2017 were 12,165 (7091 male) and 12,439 (6899 male), respectively, for a total of 24,604. Thus, head and neck trauma admissions, being *n* = 628 for the entire period, represented 2.55% percent of all trauma admissions for the period under study. These figures were extracted from the MUHAS care registry for cases deemed most likely to involve trauma. However the authors caution that these institution-wide data are indicative only. It was not possible to validate whether the care registry figures truly represented all, or even partially, the cases of trauma admitted at MUHAS.

### Strengths and Limitations

The data were derived from the Muhimbili National Hospital, which is a research-oriented teaching facility, and a national referral hospital in Tanzania. Thus, data, which are represented here, have a high degree of reliability, and are representative of trauma cases for Dar es Salaam city and the region. One common limitation to clinically derived data is that they do not typically reveal information on the circumstances and the context surrounding the injury event. All data collection serves a specific purpose. In the case of clinical data, these data are often collected with the specific aims of diagnosing patient conditions and assessing proper treatments, and such data also forms a basis for follow-up care.

In the vast majority of clinical settings around the world, the collection of environmental and contextual factors related with trauma are not routinely collected, nor are patients who are admitted to a trauma unit reliably in a position to describe the events which led to their admission. Thus clinical data in isolation (i.e., without corroborated police reports, eye witness accounts, and community-based surveillance) are routinely acknowledged for being poorly suited to address prevention as they cannot provide adequate context to demonstrate injury mechanisms in a causal fashion. This problem is exacerbated in the context of low and many middle-income countries where the routine collection of health data in general (whether clinical or otherwise) is often fraught with error, misclassified or simply not recorded at all. Thus, it remains an elusive challenge to determine risk factors for prevention. Two potential solutions to the limitations of clinical data when conducing injury research are data linkage with law enforcement, and social and verbal autopsies with the aim of reconstructing the event resulting in injury or death [21].

## 5. Conclusions

Head and neck trauma requires a multidisciplinary approach not only in treatment but also where prevention planning is concerned. A first step in the preventive process lies in understanding the burden of the condition and which groups are at increased risk. Healthcare professionals should be acquainted with such differences in injury burdens within populations to inform the development of targeted strategies for not only prevention but for improved frameworks for planning care which may ultimately lead to improvements in patient care. Subsequent to burden assessment lies a causal epidemiological examination of contributing factors and mechanisms. Future work on head and neck trauma in Tanzania should aim to clarify injury mechanisms and other relevant demographic factors which have been shown to modify risk for head and neck trauma. 

## Figures and Tables

**Table 1:** Age and sex distributions of the five leading head and neck trauma diagnoses by ICD-10 code.

ICD Code	Sex	Num. Cases	Mean Age	SD	Age (Min)	Age (Max)
S02	Female	38	22.9	17.2	0.1	70
S02	Male	239	28.9	10.2	0.1	65
S05	Female	24	31.6	28.3	1	90
S05	Male	90	21.8	15.0	0.8	62
S09	Female	21	34.6	24.6	1	71
S09	Male	56	29.3	14.8	0.1	85
S09.9	Female	16	35.8	23.0	2	77
S09.9	Male	34	32.0	15.5	2	85
S01	Female	8	24.1	12.9	0.1	44
S01	Male	36	29.1	16.3	0.1	60

**Table 2:** Breakdown of head and neck trauma by age group for cases admitted to the Muhimbili National Hospital in Dar es Salaam in 2016 and 2017.

Age (Years)	Count	Percent
0.1–5	61	9.7
6–10	32	5.1
11–15	25	4
16–20	55	8.7
21–25	90	14.3
26–30	123	19.6
31–35	87	13.9
36–40	52	8.3
41–45	34	5.4
46–50	24	3.8
51–55	13	2.1
56–60	8	1.3
60–65	6	1
66–70	7	1.1
71–75	4	0.6
76–80	2	0.3
81–85	3	0.5
86–90	2	0.3
TOTAL	628	100

**Table 3:** All head and neck trauma diagnoses by sex from 2016 and 2017 by ICD-10 code.

ICD Code	Females	Males	Total (Row)
S00	1	7	8
S01	8	36	44
S02	38	239	277
S03	1	1	2
S04	2	11	13
S05	24	90	114
S05.9	2	7	9
S06	5	19	24
S08	0	1	1
S09	21	56	77
S09.9	16	34	50
S11	0	3	3
S19	0	6	6
Total (column)	118	510	628
